# Influence of Days after Calving and Thermal Stress on the Efficacy of a Progesterone-Based Treatment in Acyclic Italian Mediterranean Buffalo

**DOI:** 10.3390/ani11113166

**Published:** 2021-11-05

**Authors:** Roberta Matera, Alessio Cotticelli, Angela Salzano, Nadia Piscopo, Anna Balestrieri, Giuseppe Campanile, Gianluca Neglia

**Affiliations:** 1 Department of Veterinary Medicine and Animal Production (DMVPA), University of Naples Federico II, 80137 Naples, Italy; roberta.matera@unina.it (R.M.); alessio.cotticelli@unina.it (A.C.); angela.salzano@unina.it (A.S.); nadia.piscopo@unina.it (N.P.); giucampa@unina.it (G.C.); neglia@unina.it (G.N.); 2 Department of Animal Health, Istituto Zooprofilattico Sperimentale del Mezzogiorno, 80055 Portici, Italy

**Keywords:** Mediterranean buffalo, progesterone, anoestrus

## Abstract

**Simple Summary:**

“Mozzarella di Bufala Campana DOP” (mozzarella cheese) is mainly produced and marketed during the spring and summer months. The buffalo is a seasonal species that increases its reproductive activity when daylight hours decrease. Therefore, to increase milk production in the favourable period, the so-called “Out of Breeding Season Mating” technique is applied. It consists of the interruption of sexual promiscuity during the naturally occurring breeding season and concentrating calving and milk production during periods of increasing daylight length. However, the application of this technique increases the incidence of anoestrus, as animals are forced to breed outside of their natural and favoured period of the year, although other factors can also increase the incidence of anoestrus, such as climate. A reduction of seasonal anoestrus can be achieved by using some hormonal treatments. In this study, primiparous acyclic buffaloes were selected and divided into three classes according to their days in milk. Animals were synchronized using P_4_ vaginal implants, and artificial insemination (AI) was performed according to protocol. The temperature–humidity index (THI) was recorded to evaluate its influence on anoestrus. Statistical analysis showed that the implemented P_4_-based treatments were highly effective in removing the anoestrus condition in buffaloes. On the contrary, no influence of the THI on the efficacy of the P_4_ synchronization treatment was observed.

**Abstract:**

The aim of this study was to evaluate the efficacy of a progesterone-based treatment on anoestrus in buffaloes. Primiparous acyclic buffaloes (*n* = 276), were divided into three classes according to their days in milk (DIM): from 50 to 90 (Class I; *n* = 86), from 91 to 150 (Class II; *n* = 102) and from 150 to 200 (Class III; *n* = 88). Animals were synchronized using P_4_ vaginal implants, followed by timed artificial insemination (TAI). They were then allowed to enter into a larger group of buffaloes for natural mating 15 days after AI was performed, and pregnancy status was monitored from then on at 15-day intervals. Finally, the temperature–humidity index (THI) was calculated. Statistical analysis was performed by ANOVA by means and both multiple and linear regression. The total pregnancy rate (PR) was 87.7%, with no differences among DIM classes (88.0, 92.4, and 80.0% in Classes I, II, and III, respectively). However, the PR at TAI tended to be higher (*p* = 0.07) in buffaloes in Class II. The follicle (FL) area in Class II buffaloes was larger (*p* < 0.01) than that of the other classes. No influence of the THI on the total PR was recorded. The pregnancy outcome at TAI was affected by the FL area (odds ratio = 2.237; *p* < 0.05) and body condition score (BCS) (odds ratio = 1.256; *p* < 0.05). In conclusion, treatment with vaginal P_4_ optimizes pregnancy rates in anoestrus buffaloes, particularly when the animals are in mid-lactation and show an optimal BCS. Furthermore, the THI does not seem to affect the efficiency of the progesterone treatment.

## 1. Introduction

The buffalo species is an important livestock resource in both developing and developed countries [1,2], as demonstrated by the worldwide increase in the population in the last decades [3]. Although it is used in many countries for draught power and meat production, in developed countries, milk production is the main reason for buffalo farming. In Italy, the breeding strategies applied on buffalo farms have reached a high grade of innovation and mechanization, similarly to modern dairy cattle farms. In fact, according to the statistics of the Italian Association of Buffalo Breeders (ANASB), in the last several years, the average reported yield in Italian Mediterranean buffalo was 2368 kg of milk/270 days of lactation, and the fat and protein percentages were 7.77% and 4.64%, respectively [4]. 

In order to guarantee optimal milk production and ensure economic farm profitability, particular attention should be paid to the optimization of reproductive efficiency [3,5]. In Italy, buffalo milk is entirely used for the production of mozzarella cheese, which, in the late 90s, received important recognition by being included in the list of national DOP (Protected Denomination of Origin) products, and it is marketed mainly during the spring–summer period. Therefore, in order to achieve most of the mozzarella milk production during the desired marketing period, the “Out of Breeding Season Mating” (OBSM) technique [6] must be applied. It consists in the interruption of sexual promiscuity (or performed AI) during the breeding season (when daylight length decreases), with the goal of concentrating calving and milk production during the remaining periods of increasing daylight length [7]. In fact, although heats and ovarian activity can be observed and monitored in some animals throughout the year, the buffalo at our latitudes are tendentially “short day” breeders [2], resulting in higher reproductive activity when daylight hours decrease. Such tendential seasonality may be derived from its origin as animal species in the Indo Valley region, where forage availability is recorded during periods of decreasing daylight length [5,6]. Indeed, as observed in other seasonal species, anoestrus and embryonic mortality in buffaloes are the main reproductive problems reported during the period of the year characterized by increasing daylight hours [8].

The application of OBSM increases the incidence of anoestrus, as animals are forced to breed outside of their natural period. In recent studies, prolonged post-partum anoestrus in buffaloes calving during periods of increasing daylight hours was reported [9], probably due to low pulsatile secretion of Luteinizing Hormone (LH) that cannot guarantee appropriate follicle development [10]. Although it is mainly due to increasing daylight length, other factors can also increase the incidence of anoestrus, such as climate, parity, and farm management [9]. Several studies have been carried out to elucidate the hormonal basis of anoestrus [5,9], although little and contrasting information is actually available on the influence of other variables, such as days in milk (DIM) and the temperature-humidity index (THI), on productive and reproductive performance in buffalo [11,12]. In particular, buffaloes are able to adapt to different environmental conditions, from tropical to temperate environments, and the response to thermal stress is quite variable (for review, see [12]). According to several authors, buffalo are considered more tolerant to heat stress than cattle [13], although high relative humidity and direct solar radiations may severely affect their performance. A mean THI value of 73 for 20 days before breeding was demonstrated to have a deleterious effect on the conception rate in dairy cattle [14], while no information is available on the impact of heat stress on reproduction, particularly during synchronization protocols, in buffalo.

A reduction of seasonal anoestrus can be achieved using some hormonal treatments [15,16,17]. Significant results have been recorded using either intravaginal progesterone (P_4_) devices [15,18] or norgestomet ear implants [19], which are associated with prostaglandin and equine chorionic gonadotrophin (eCG). However, little information is available on the efficacy of progesterone devices in buffaloes with different days open and maintained in the same conditions with the subsequent resumption of the ovarian cycle and pregnancy rate (PR). Therefore, the aim of this study was to assess the influence of thermal stress and days after calving on progesterone-based treatment in acyclic buffaloes undergoing TAI and natural mating.

## 2. Materials and Methods

### 2.1. Farm

The Ethical Animal Care and Use Committee of the Federico II University of Naples approved the experimental design of the study (protocol no. 996072017). The trial was carried out on a commercial buffalo farm located in the South of Italy between 39.0°N and 41.5°N, where about 900 buffaloes were bred. Lactating cows received a total mixed ration administered twice daily. The detailed composition of the diet and the chemical nutritional value are reported in Table 1. The ration was formulated to meet the nutritional requirements for maintenance and production according to previous studies [20]. A total of 276 primiparous adult buffaloes, weighing about 550 kg and showing an optimal body condition score (between 7.0 and 8.5 on a 1 to 9 scale with 0.25 increments [21]) were included in the study. The average BCS was 7.8 ± 0.02, and the average milk yield was 12.1 ± 0.2 kg.

### 2.2. Reproductive Management

Reproductive management was carried out by means of both artificial insemination (AI) and natural mating. Adult buffalo cows were maintained in groups of 60 animals in cement open yards that allowed 15 m^2^/head. The OBSM technique was implemented on the farm; thus, mating was allowed from February to September (8 months) in order to centre most of the deliveries and, consequently, the maximum milk production from January to August. Immediately (within 3–5 days) after calving and within 20 days post-partum, the buffaloes underwent gynaecological examinations and assessments of the reproductive tract (Control Genital Apparatus—CGA) to assess the conditions of the apparatus. A voluntary waiting period (VWP) of 40 days was maintained. Following the VWP, natural mating was allowed and guaranteed by the presence of four bulls in each group (1 bull for every 15 cows). Gynaecological examinations of the animals were carried out 7 days apart by means of ultrasound to assess the ovarian and uterine status. Animals found to be affected by other conditions (mastitis, lameness, etc.) were excluded from the study. Furthermore, if the animals did not show corpora lutea and/or follicles larger than 0.7 cm following three consecutive examinations, they were considered to be in anoestrus and were subjected to oestrus synchronization [17] and AI (see below). On the contrary, if a pregnancy was recorded, the diagnosis was confirmed at least two times 30 days apart, and the animals were excluded from further investigations.

### 2.3. Experimental Design and Selection of the Animals

Only non-cyclic primiparous lactating buffaloes were selected from a larger number of animals during a period of increasing daylight length (from April to July). Following VWP, animals underwent gynaecological examinations 7 days apart; if no corpora lutea or large follicles were detected, two further examinations were carried out to confirm the anoestrus condition. Therefore, the selection criteria were: (i) absence of corpus luteum and/or follicles larger than 0.7 cm in at least three consecutive clinical examinations by ultrasound 7 days apart, (ii) no clinically evident inflammatory condition of the uterus, and (iii) a BCS between 7 and 8.5. If these characteristics were met, the animals were entered into a synchronization treatment based on a progesterone intravaginal device and AI (see below). A paillette from the same lot to be used for AI was analysed under a light microscope to assess sperm progressive motility and the live spermatozoa percentage. Fifteen days after AI, inseminated buffaloes were included in a larger group for natural mating. Pregnancy diagnosis was carried out 27 days after AI and confirmed on days 45 and 90 after AI; if the buffaloes were not pregnant, they underwent clinical examinations for pregnancy diagnosis every 15 days. According to pregnancy, animals were classified as pregnant at TAI or pregnant at the 1°, 2°, and 3° oestrous cycle post-insemination. Finally, data on temperature and humidity were collected on the first day of the synchronization treatment and the day of TAI to calculate the THI and the differences between the initial and final THI (ΔTHI). 

### 2.4. Synchronization Treatment and TAI

**Figure 1:**
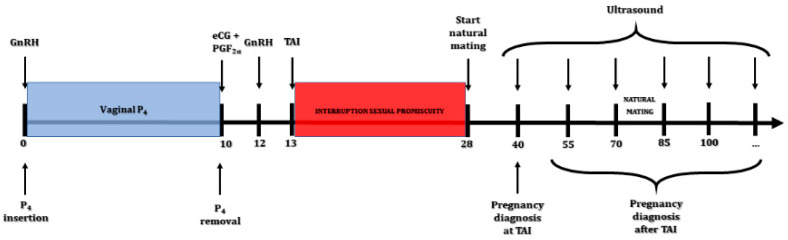
Synchronization treatment and experimental design during the study.

Acyclic buffaloes were synchronized by means of a P_4_-based synchronization treatment (Figure 1). Briefly, selected buffaloes received a GnRH agonist (buserelin acetate, 12 µg; Receptal^®^; Intervet, Milan, Italy) on day 0 together with the insertion of a 1.38 g progesterone intravaginal device (CIDR^®^; Zoetis SRL, Rome, Italy). After 10 days, the device was removed, and the animals received a luteolytic dose of cloprostenol (500 mg of Estrumate^®^; MSD Animal Health, Milan, Italy) together with 750 IU of PMSG (Folligon; Intervet MSD, Milan, Italy). Forty-eight hours later, a further GnRH agonist was administered to ensure the synchronization of ovulation. Timed artificial insemination (TAI) was performed 60 h after prostaglandin administration and 16 h after the last GnRH administration. On the day of AI, the animals underwent ultrasound examination (MyLab 30Gold^®^; Esaote, Genova, Italy), and the size of the preovulatory follicle was recorded. The area of the preovulatory follicle was calculated as previously described [22]:

(1) $$\mathrm{FL}\;\mathrm{area}=\frac{a}{2}\times \frac{b}{2}\times \pi$$

where:

✓ FL = follicle
✓ a = major axis of the follicle
✓ b = minor axis of the follicle
✓ π=3.14

TAI was carried out by the same experienced veterinarian using frozen and thawed semen of one buffalo bull.

### 2.5. Meteorological Information

The environmental temperature and relative humidity (both on an hourly basis) were recorded at an official weather station (Davis Vantage Pro 2 plus wireless, Hayward, CA, USA) located 1 km from the farm. Data were collected daily and used to calculate the average temperature–humidity index (THI) according to the following formula [23]:

THI = (0.8 × T) + (RH/100) × (T − 14.4) + 46.4(2)

where:

✓ T is the environmental temperature in °C
✓ The relative humidity (RH) in %. The daily average RH value was calculated using the arithmetic mean and included in the equation

Data on environmental minimum temperature, maximum temperature, thermal excursion, and relative humidity were recorded. THI values recorded at the start of the synchronization protocol (THI_start_) and the end of the synchronization protocol (THI_end_) were used to calculate the average during the period of synchronization (THI_mean_) and the excursion throughout the period of synchronization (ΔTHI).

### 2.6. Statistical Analysis

Statistical analyses were performed using SPSS (28.0) for Windows 10 (SPSS Inc., Chicago, IL, USA). Buffaloes were assigned to three classes according to their days in milk (DIM): from 50 to 90 DIM (catabolic phase of lactation; *n* = 86), from 91 to 150 DIM (first phase of the anabolic phase of lactation; *n* = 102), and from 150 to 200 DIM (second phase of the anabolic phase of lactation; *n* = 88). Moreover, according to the THI_mean_ values, buffaloes were divided into four classes: Class A (THI < 63), Class B (THI between 63.1 and 72), Class C (THI between 72.1 and 75), and Class D (THI > 75.1). Pregnancy rates among post-synchronization oestrus cycles (I, II, III, IV, and +IV), among DIM classes (I, II, and III), and among different months were compared using nonparametric tests (Pearson and Χ-square). Differences between groups were evaluated by Analysis of Variance (ANOVA). A General Linear Model (GLM) procedure was utilized to assess the influence of THI_start_, THI_end_, THI_mean_, and ΔTHI on the follicle area at TAI by multivariate ANOVA. BCS, DIM, month of calving, and their interaction were included in the model. All data are reported as means ± standard error. A difference of *p* < 0.05 was accepted as statistically significant. 

The relationship between the DIM, BCS, THI, follicular area, and treatment–conception interval was studied using multiple linear regression (stepwise procedure). Finally, the odds ratio for the pregnancy outcome at TAI was assessed by multiple logistic regression (stepwise procedure). Days in milk, milk yield (MY), BCS, follicle area, month of calving, THI_start_, THI_end_, THI_mean_, and ΔTHI were included in the model as continuous variables.

## 3. Results

At the end of the mating period, an overall pregnancy rate of 87.7% was recorded (Table 2). In particular, more than 50% and 35% of the animals were pregnant after AI and within 3 oestrus cycles after AI, respectively (Table 2). 

**Figure 2:**
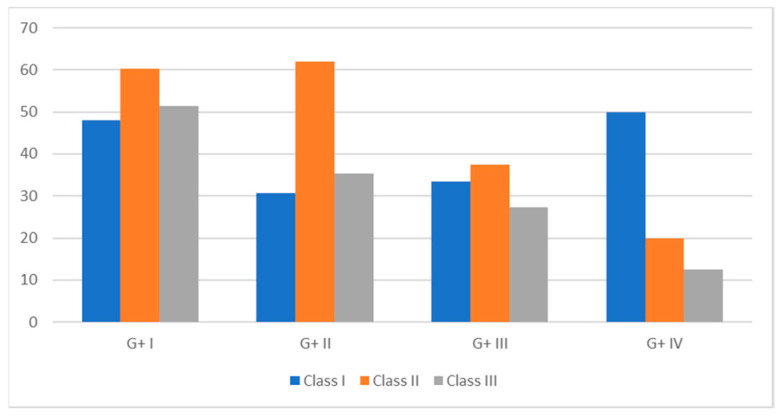
Pregnancy rate recorded throughout the study in buffaloes with different days in milk (Class I: <90 DIM; Class II: 91–150 DIM; Class III: >151 DIM).

No differences were recorded in the total pregnancy rate when the buffaloes were classified according to their days in milk at the start of the treatment (88.0, 92.4, and 80.0% PR in Classes I, II and III, respectively). However, the number of pregnant buffaloes at TAI in Class II (91–150 DIM) tended to be higher (*p* = 0.07) compared with those in Class I (60.4 and 48.0 in Classes II and I, respectively). Furthermore, a significantly (*p* < 0.01) higher pregnancy rate was observed in Class II in the first cycle post-insemination compared with the others (Figure 2). The BCS was significantly higher (*p* < 0.01) in buffaloes in Classes II and III compared with those in Class I (7.60 ± 0.1, 7.97 ± 0.1, and 8.07 ± 0.1 in Classes I, II, and III, respectively).

Excluding buffaloes pregnant at TAI, the mean interval between the end of treatment and conception was significantly (*p* < 0.05) higher for animals in Class I compared with those in Classes II and III (48.2 ± 4.1 days in Class I vs. 27.5 ± 2.4 and 34.6 ± 4.7 days in Classes II and III, respectively).

The follicle area recorded at TAI was significantly (*p* < 0.01) larger in buffaloes in Class II compared with those in other classes (Table 3). Although no differences were recorded between pregnant and non-pregnant buffaloes within each class, buffaloes pregnant at TAI had significantly larger follicles (*p* < 0.01) than their non-pregnant counterparts (Table 3).

**Figure 3:**
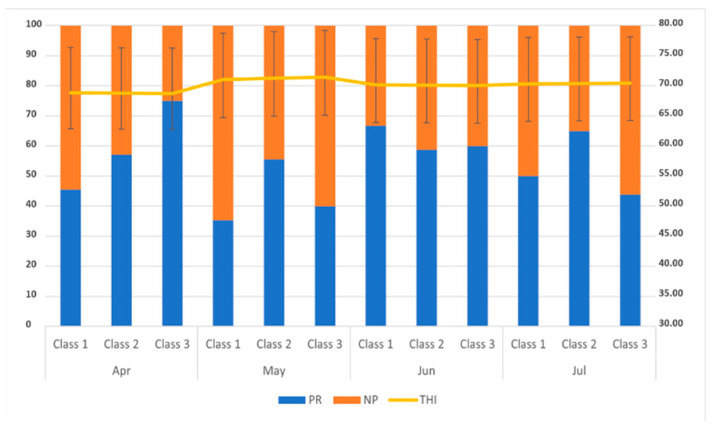
Pregnancy risk recorded in different months in buffaloes with different days in milk (Class I: <90 DIM; Class II: 91–150 DIM; Class III: >151 DIM) and THI values recorded throughout the study.

A further analysis was carried out to evaluate the pregnancy rate (PR) in animals treated throughout the year (Figure 3). Few differences were observed throughout the months. However, a higher (*p* < 0.05) PR was recorded in buffaloes treated in June compared with those treated in July. Similarly, the PR at TAI and in the first cycle post-insemination was significantly (*p* < 0.05) higher in June than in May (Figure 3). 

The average THI throughout the study was 70.2 ± 0.42. No influence of THI on the total pregnancy rate was recorded. In particular, when the animals were divided into the four classes of THI, pregnancy rates of 43.3, 52.2, 59.7, and 57.5 were recorded in Classes A, B, C, and D, respectively. Furthermore, the multiple linear regression analysis only showed a significant (*p* < 0.01) influence of BCS at TAI on the follicular area (r^2^ = 0.242), according to the following equation:

FL_area_ = −0.299 + (0.255 × BCS) (3)

Additionally, in this case, no influence of THI was recorded. Finally, the multiple logistic regression analysis (Table 4) showed that the pregnancy outcome at TAI was affected by the FL area (odds ratio = 2.237; *p* < 0.05) and BCS at TAI (odds ratio = 1.256; *p* < 0.05), while no influences of other variables were recorded.

## 4. Discussion

Anoestrus is a naturally occurring event in buffaloes during the out-of-breeding season, which coincides with the time of year characterized by increasing day length. In Italy, its incidence is particularly high when the OBSM technique is implemented, thus affecting the reproductive efficiency of the herd. In this study, we evaluated the influence of DIM and THI on exogenous progesterone administration and synchronization in buffaloes in intensive conditions after TAI and subsequent natural mating. In our study, P_4_-based treatment was able to guarantee both an optimal conception rate and the resumption of ovarian activity, leading to a pregnancy rate of more than 87% at the end of the breeding season. These results are in agreement with previous trials carried out in the buffalo species in which treatment with exogenous progesterone guaranteed the removal of seasonal anoestrus, achieving a satisfactory pregnancy rate [15,18]. According to several studies (for review, see [9]), the anoestrus condition in the buffalo species is probably due to the low secretion of LH. It is largely known that LH RNA levels are increased in rat pituitary cells in vitro following P_4_ action, probably via GnRH receptor regulation [24]. In fact, in vivo treatment by exogenous progesterone reduces the pulsatile secretion of GnRH [25] and, consequently, the synthesis of GnRH receptors [26]. This causes a reduced sensitivity of the pituitary gland to GnRH. Furthermore, an increase in LH and FSH was observed during P_4_ treatment in ovariectomized and hypothalamic–pituitary-disconnected ewes [27] due to augmented synthesis and inhibition of secretion. Similarly, P_4_-based treatments have been demonstrated to increase the pulsatile secretion of LH during and after treatment in cattle [28], favouring follicular growth and oestradiol concentration; this in turn leads to a higher number of LH receptors of granulosa and theca cells and, consequently, ovulation [29]. It is likely that this process is also the basis of the removal of seasonal anoestrus in buffalo species.

It is also worth pointing out that a PR of more than 94% was recorded within the second cycle after TAI. In a previous trial carried out on buffalo heifers, the efficiencies of two re-synchronization treatments throughout the year were compared [18]. Although in this case, buffaloes were mated through TAI, a pregnancy rate of about 90% was achieved within the third cycle when synchronization was performed with a P_4_-vaginal implant. This interesting result confirms our finding identifying a high fertile condition in buffalo in the first cycles after synchronization. 

According to several studies, a low pregnancy rate (from 7 to 30%) has been recorded out of the breeding season in buffalo (for review, see [9]) due to the high incidence of anoestrus. This is the first study evaluating the influence of the days in milk on the efficacy of synchronization programs with P_4_ vaginal implants in buffaloes. However, the PR after TAI and in the first subsequent cycle in Class II buffaloes tended to be higher than that recorded in Class I. Furthermore, it is worth pointing out that buffaloes in Class II showed larger preovulatory follicles at TAI compared with their Class I counterparts. It is known that buffaloes in Class I experienced a typical post-partum anoestrus, previously described in cattle [30] and buffalo [31]. This is not influenced by the calving season but probably by the negative energy balance that the animals encounter during early lactation. This condition significantly affects both the resumption of ovarian activity and preovulatory follicle growth, impairing reproductive efficiency. This interesting finding is further confirmed by the lower BCS recorded in buffaloes in Class I compared with those in Class II. Several authors have demonstrated that low BCS is associated with a low pregnancy rate in both cattle [32] and buffalo [31]. This is probably due to the utilization of nutrients for metabolism maintenance rather than for reproduction [33]. On the contrary, a typical seasonal (or summer) anoestrus has been recorded in buffaloes in Class II [6,12]. In a natural environment, this is a physiological condition that avoids mating and, consequently, calving in periods in which the environmental conditions and availability of forage would not be sufficient to ensure the survival of the offspring and the conservation of the species. Usually, in these cases, a great proportion of buffaloes resume their cyclic ovarian activity only in coincidence with the following breeding season when the animals are exposed to periods of decreasing daylight length [9].

Pregnant buffaloes also showed a larger preovulatory follicle compared with their non-pregnant counterparts. It is known that follicular growth is stimulated by the pulsatile secretion of gonadotrophin [34], but in anoestrus animals, it is guaranteed by eCG administration [35], which has both FSH and LH activity. Monteiro et al. [36] also demonstrated a high correlation between the size of the dominant follicle at TAI and both ovulation and pregnancy rate at 45 days post-AI. Furthermore, larger follicles were also associated with larger corpora lutea and augmented progesterone secretion [22], reducing the incidence of embryonic mortality [8].

Little information is available on the influence of the THI on productive and reproductive performance in buffaloes [37]. Several authors agree on the high thermo-tolerance of buffaloes to hot and humid climates, which is due to several factors, such as the presence of melanin pigments in the epidermis to prevent the penetration of the dermis by ultraviolet rays and a high number of sebaceous glands [38]. However, according to some recent studies [37], it has been demonstrated that THI values higher than 82 cause a decline in milk yield of about 1%. Similarly, a reduced conception rate after AI from 4 to 21% has been recorded in three buffalo genotypes in Egypt together with an increase in THI values. In our study, no influence of THI was recorded on follicular growth or BCS. Furthermore, according to the multiple regression analysis, the THI also did not influence the PR at TAI, while strong effects of the FL area and BCS were recorded. It is worth pointing out that the THI values recorded in this trial rarely reached 80, and no differences were recorded between the THI at the start of the synchronization protocol and that at the end. It is known that a deleterious effect of the THI is observed from a THI higher than 72 in dairy cows (for review, see [39]) and higher than 75 in buffalo [37]. In our study, a THI_mean_ higher than 75 was recorded in only 29.7% of cases, and it never exceeded 80; it cannot be ruled out that the low number of animals affected this result, as demonstrated by the evidence that buffaloes that encounter a THI higher than 75 showed a similar PR to the others. 

## 5. Conclusions

In conclusion, this study demonstrated that a P_4_-based treatment is highly effective in removing the anoestrus condition in buffaloes, allowing a PR higher than 87% within the third oestrus cycle after treatment. According to our results, DIM does not seem to influence PR results, with the exception of a trend for a higher PR recorded in Class II, which represents animals in a positive energy balance (after 91 days of lactation). This is probably due to both the larger size of the preovulatory follicle and the optimal BCS. On the contrary, no influence of THI on the efficacy of the synchronization treatment was observed. 

## Figures and Tables

**Table 1:** Composition (kg), chemical characterization (% of dry matter, DM), and energy density (Milk Forage Unit—MFU) of the diet utilized during the trial.

Feed	Kg
Maize silage	20.0
Brewers grains	8.0
Alfalfa Hay	4.4
Straw	1.0
Corn flakes	3.1
Soybean meal	2.1
Hydrogenated fat	0.25
Calcium carbonate	0.08
Sodium bicarbonate	0.06
Chemical Composition	
DM (kg)	16.82
CP (%/DM)	14.80
EE (%/DM)	4.85
CF (%/DM)	19.51
NDF (%/DM)	41.09
ADF (%/DM)	23.68
Ashes (%/DM)	5.62
NSC (%/DM)	32.03
Starch (%/DM)	21.66
Calcium (%/DM)	0.84
Phosphorus (%/DM)	0.45
MFU (%/DM)	0.92

CP = crude protein; EE = ether extract; CF = Crude fibre; NDF = neutral detergent fibre; ADF = acid detergent fibre; ADL = acid detergent lignin; NSC = non-structural carbohydrates.

**Table 2:** Pregnancy rate recorded throughout the study in buffaloes that underwent TAI and natural mating.

ANIMALS	TAI	Cycles Post-Insemination			TOTAL
		1°	2°	3°	
Buffaloes inseminated (*n*)	276	128	74	50	276
Number pregnant (*n*)	148	54	24	16	242
Percentage pregnant (%)	53.6 ^a^	42.2 ^b^	32.4 ^b^	32.0 ^b^	87.7
Percentage of total pregnant (%)	61.2	22.3	9.9	6.6	100

Values in the same rows with different superscripts are significantly different (^a,b^, *p* < 0.05).

**Table 3:** Area of the dominant follicle recorded on the day of TAI in pregnant and non-pregnant buffaloes with different days in milk (Class I: <90 DIM; Class II: 91-150 DIM; Class III: >151 DIM).

CLASS	PR TAI	NP TAI	TOTAL
Class I	1.24 ± 0.0 ^X^	1.14 ± 0.0 ^X^	1.19 ± 0.0 ^X^
Class II	1.59 ± 0.0 ^Y^	1.49 ± 0.0 ^Y^	1.55 ± 0.0 ^Y^
Class III	1.23 ± 0.0 ^X^	1.16 ± 0.0 ^X^	1.22 ± 0.0 ^X^
TOTAL	1.39 ± 0.0 ^A^	1.25 ± 0.0 ^B^	1.34 ± 0.0

Values are expressed as mean ± standard error. ^X,Y^, values within the same column differ significantly at *p* < 0.01. ^A,B^, values within each row are significantly different at *p* < 0.01.

**Table 4:** Results (odds ratios and 95% confidence intervals) of the logistic regression analysis for the risk of pregnancy at timed artificial insemination (TAI), considering several factors: days in milk (DIM); body condition score (BCS); area of the preovulatory follicle (FL area); month of calving (month_calv_); milk yield (MY); and the THI recorded at the start (THI_start_), at the end (THI_end_), and during (THI_mean_) the trial. Data on THI excursion during the synchronization period were also considered (ΔTHI).

Variable	Coefficient	Odds Ratio	95% Conf. Int.	*p* Value
Constant	−2.427	0.0883	64.346	0.470
DIM	0.149	1.161	1.808	0.509
BCS	0.101	1.215	2.312	0.031
FL area	0.698	2.010	3.794	0.014
Month_calv_	0.189	1.208	1.804	0.354
MY	0.0424	1.043	1.142	0.291
THI_start_,	−0.1000	0.905	0.974	0.215
THI_end_,	0.0601	1.062	1.158	0.394
THI_mean_	0.140	1.150	1.370	0.156
ΔTHI	−0.0304	0.970	1.026	0.284

## Data Availability

The data that support the findings of this study are available from the corresponding author, upon reasonable request.
